# Pivotal roles of phyllosphere microorganisms at the interface between plant functioning and atmospheric trace gas dynamics

**DOI:** 10.3389/fmicb.2015.00486

**Published:** 2015-05-22

**Authors:** Françoise Bringel, Ivan Couée

**Affiliations:** ^1^Laboratory of Molecular Genetics, Genomics, and Microbiology, Université de Strasbourg/CNRS, UNISTRA UMR 7156Strasbourg, France; ^2^Ecosystems-Biodiversity-Evolution, Université de Rennes 1/CNRS, UMR 6553Rennes, France

**Keywords:** plant–microorganism interactions, aerial plant organs, environmental genomics, volatile organic compounds, phyllosphere–atmosphere interface, global change

## Abstract

The phyllosphere, which *lato sensu* consists of the aerial parts of plants, and therefore primarily, of the set of photosynthetic leaves, is one of the most prevalent microbial habitats on earth. Phyllosphere microbiota are related to original and specific processes at the interface between plants, microorganisms and the atmosphere. Recent –omics studies have opened fascinating opportunities for characterizing the spatio-temporal structure of phyllosphere microbial communities in relation with structural, functional, and ecological properties of host plants, and with physico-chemical properties of the environment, such as climate dynamics and trace gas composition of the surrounding atmosphere. This review will analyze recent advances, especially those resulting from environmental genomics, and how this novel knowledge has revealed the extent of the ecosystemic impact of the phyllosphere at the interface between plants and atmosphere.

Highlights

• The phyllosphere is one of the most prevalent microbial habitats on earth.

• Phyllosphere microbiota colonize extreme, stressful, and changing environments.

• Plants, phyllosphere microbiota and the atmosphere present a dynamic continuum.

• Phyllosphere microbiota interact with the dynamics of volatile organic compounds and atmospheric trace gasses.

## Introduction

Microbial communities on or around plants play a major role in plant functioning and vigor. Rhizospheric microbial communities, associated with root systems, have been extensively studied and best characterized, as they have been shown to be directly involved in crop productivity through their roles in bioaccessibility of mineral nutrients, protection against pathogens and release of phytohormones to stimulate plant growth. However, the phyllosphere, which *lato sensu* consists of the aerial parts of plants, and therefore primarily, of the set of photosynthetic leaves, is one of the most prevalent microbial habitats on earth.

The total extent of lower and upper surfaces of leaves is thought to represent 10^9^ km^2^ that could harbor 10^26^ bacterial cells (Vorholt, 2012) and is a major potential entrance for phytopathogenic organisms, whose colonization of the plant must not only overcome plant defenses, but also confront competition from existing microorganisms. Although their numbers are much lower than those of bacteria, phyllosphere-associated fungi are potentially involved in major ecophysiological functions, such as interactions with pathogenic fungi, C/N dynamics or the initial steps of leaf litter degradation (Voříšková and Baldrian, 2013). As for archaea, the first studies that have investigated their occurrence suggest that they are a rather minor component of phyllospheric communities and are more present in the rhizosphere (Knief et al., 2012). Plant microbiota, and especially phyllosphere microbiota, are thus an important field of study for understanding community assemblage processes and the mechanisms of community maintenance *in natura*.

Knowledge on phyllosphere microbiota can reveal the mechanisms that govern processes at the interface between plants, microorganisms and the atmosphere, either in pristine environments, or in agricultural or anthropogenic environments. In the case of epiphytic microorganisms, which live on the surface of plant tissues, the phyllosphere is an extreme and unstable habitat, with characteristics of oligotrophy such as limitation in carbon and nitrogen nutrients, and of multiple and highly fluctuating physicochemical constraints (high light, ultraviolet radiation, temperature, dessiccation). Recent high-throughput –omics technologies have lifted a range of analytical bottlenecks, thus raising fascinating opportunities for characterizing in an exhaustive way the spatio-temporal structure of phyllosphere microbial communities in relation with the structural, functional, and ecological properties of host plants (genotype, anatomy, developmental, nutritional and physiological status, biogeography). The recent advances that have been brought to the field of microbial life in the phyllosphere, especially through the development of environmental genomics and metagenomics, have considerably expanded our understanding of the roles of phyllosphere microbial communities in plant–environment interactions and of the ecosystemic impact of the phyllosphere. Thus, major progress is expected in order to understand the impacts on the physicochemical properties of the environment, such as climate dynamics, the dynamics of numerous gaseous compounds [levels of volatile organic compounds (VOCs), gaseous plant hormones, and volatile pollutants] and the trace gas composition of the surrounding atmosphere.

## Confrontation of Microorganisms with the Extreme and Stressful Physicochemical Conditions of the Phyllosphere

**FIGURE 1:**
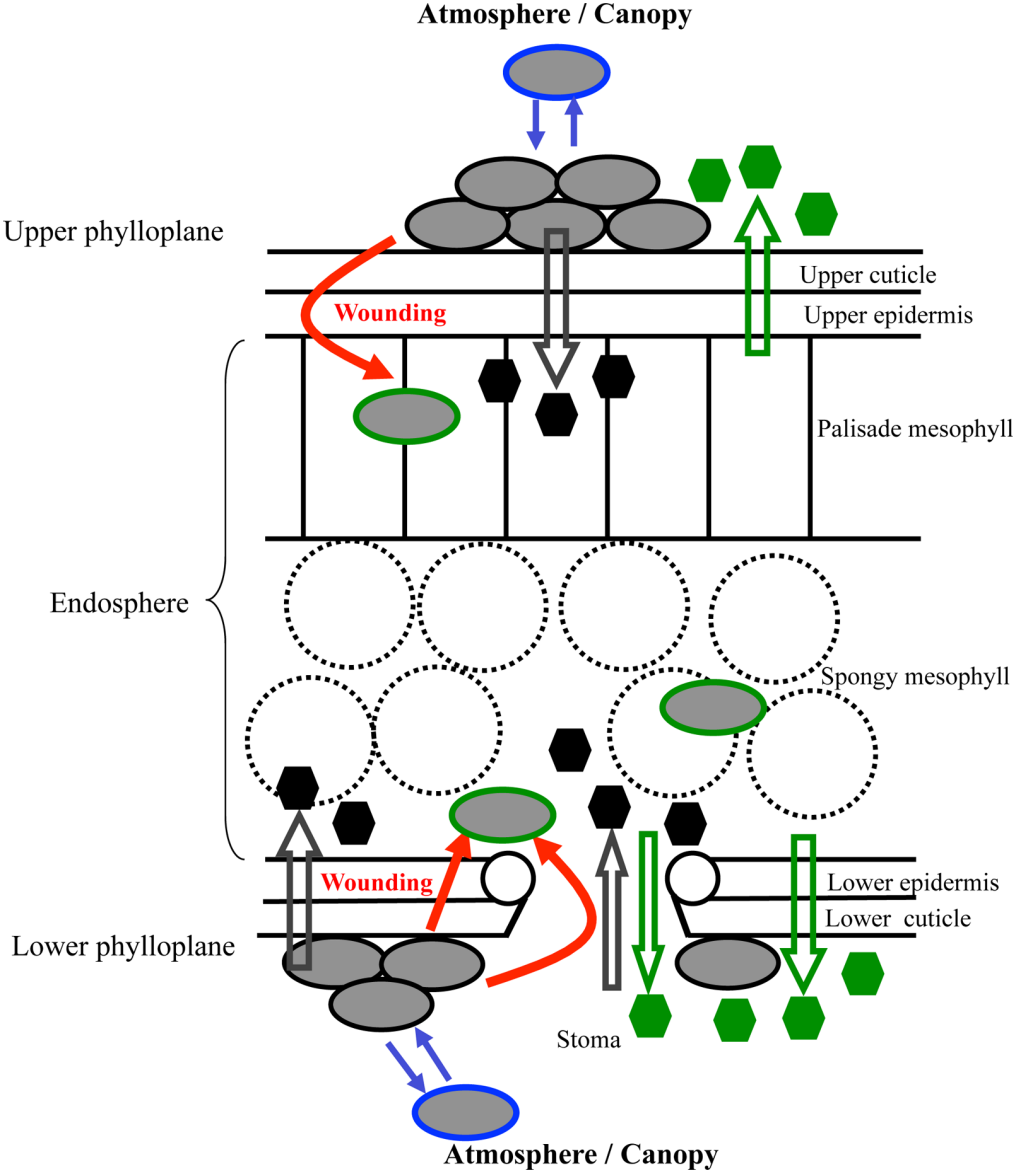
**General histological features and biochemical exchanges of the phylloplane**. The schematic leaf cross section shows interactive exchanges (solid arrows) involving epiphytic microorganisms (gray ellipsoids with black edge) or endophytic microorganisms (gray ellipsoids with green edge) and leaf structures. It also shows the potential fluxes of plant (open green arrows) and microbial (open gray arrows) chemicals (green or black hexagons) that can occur through excretion, exudation, guttation, wounding, leaching, or infiltration. Blue arrows indicate the dynamics of the epiphyte communities with sweeping of bacteria from plant leaves and colonization from airborne microorganisms (gray ellipsoids with blue edge).

Among the different above-ground portions of plants found in the phyllosphere such as the caulosphere (stems), the anthosphere (flowers) and the carposphere (fruits), the phyllophane (surface of leaves; **Figure 1**) presents many peculiar features for microbial life (Kowalchuk et al., 2010; Vorholt, 2012; Rastogi et al., 2013; Turner et al., 2013; Müller and Ruppel, 2014). Leaf surfaces are by themselves a complex architecture of microenvironments showing bidimensionally and tridimensionally heterogeneous structures. The characteristics of upper or lower phylloplane (Eglinton and Hamilton, 1967; Schreiber et al., 2004; Reisberg et al., 2013) affect the interactions between epiphytic microorganisms, which live on plant surfaces, in particular by modulating the access to nutrients from leaf tissues (Ruinen, 1961; Bulgarelli et al., 2013), by providing more or less protection from incoming sunlight (Atamna-Ismaeel et al., 2012a), or by presenting gateways for penetration within the plant endosphere (Hirano and Upper, 2000; Schreiber et al., 2004). Epiphytic microorganisms must adjust to multiple fluctuations involving the season cycle, the day/night cycle, and the developmental, morphological and anatomical dynamics of the plant, from the bud to the senescing leaf, or from the flower to the fruit. Plant photoassimilates like sucrose, fructose, and glucose are present on leaf surfaces (Trouvelot et al., 2014), but day/night fluctuations result in important modifications of the plant metabolite profile, and therefore of nutrient availability for the growth of epiphytic microorganisms. Moreover, plant metabolic status, especially carbohydrate status, is highly responsive to conditions of abiotic or biotic stresses (Couée et al., 2006; Trouvelot et al., 2014). Plant metabolites, such as soluble sugars, polyols, amino acids, amines, VOCs such as isoprenoids, halogenated compounds or alcohols, as well as plant water and salts, are not freely and directly available for epiphytic microorganisms. Plant leaf surfaces are generally protected by lipidic and waxy cuticles that greatly limit water and metabolite fluxes, and biochemical exchanges therefore depend on multiple pathways including excretion, exudation, guttation, wounding, leaching, or infiltration (**Figure 1**). All of these characteristics result in an oligotrophic habitat with limitations in carbon and nitrogen resources.

Phyllospheric microorganisms are subjected to multiple physicochemical stresses that can very rapidly vary through leaching, temperature changes, variations of sunlight-exposure, fluctuations of reactive oxygen species production and therefore of oxidative stress intensity. Trees adapted to desertic conditions can secrete soluble compounds that result in alkalization and salinization of leaf surfaces, thus generating saline or alkaline stress in phyllosphere microbes (Finkel et al., 2012). PhyR, which is a general stress response regulator necessary for plant colonization by several alpha-proteobacteria, is enhanced during growth on the phyllosphere compared to growth in liquid media in the laboratory (Gourion et al., 2006; Iguchi et al., 2013). A *ΔphyR* deletion mutant of the methanotroph *Methylosinus* sp. B4S, that colonizes *Arabidopsis* leaf surfaces, was demonstrated to be more sensitive to heat shock and ultraviolet light than the wild-type strain (Iguchi et al., 2013), thus emphasizing the importance of general stress responses for microorganisms living in the phyllosphere.

Adaptation to the phyllospheric lifestyle appears to rely on a variety of mechanisms related to a diversity of physico-chemical and biotic constraints. Epiphytic microorganisms can develop tolerance and resistance mechanisms against the antimicrobial and immunity compounds produced by plant tissues or against competing microorganisms (Trouvelot et al., 2014). Numerous studies have focused on the interactions between bacterial quorum-sensing signals and plant roots (Mathesius et al., 2003; Patel et al., 2013), while few studies have dealt with plant leaf microbial communities. Epiphytic microorganisms that display enzymes degrading *N*-acylhomoserine lactone (AHL) quorum-sensing signals have been reported in the tobacco phyllosphere (Ma et al., 2013), thus suggesting that signaling circuits may be involved in shaping complex epiphyllic microbial communities. Epiphytic microorganisms can also develop mechanisms of aggregate formation or of exopolysaccharide synthesis- in order to improve adhesion or protection from dessication (Yu et al., 1999; Monier and Lindow, 2003). Finally, they can also synthesize and secrete phytohormonal compounds, such as indole-3-acetic acid, which facilitates nutrient exudation from plant tissues as a result of plant cell wall relaxation (see details in Vorholt, 2012). However, the complete understanding of these adaptive mechanisms remains incomplete.

## Structure and Diversity of Phyllosphere Microbiota

**FIGURE 2:**
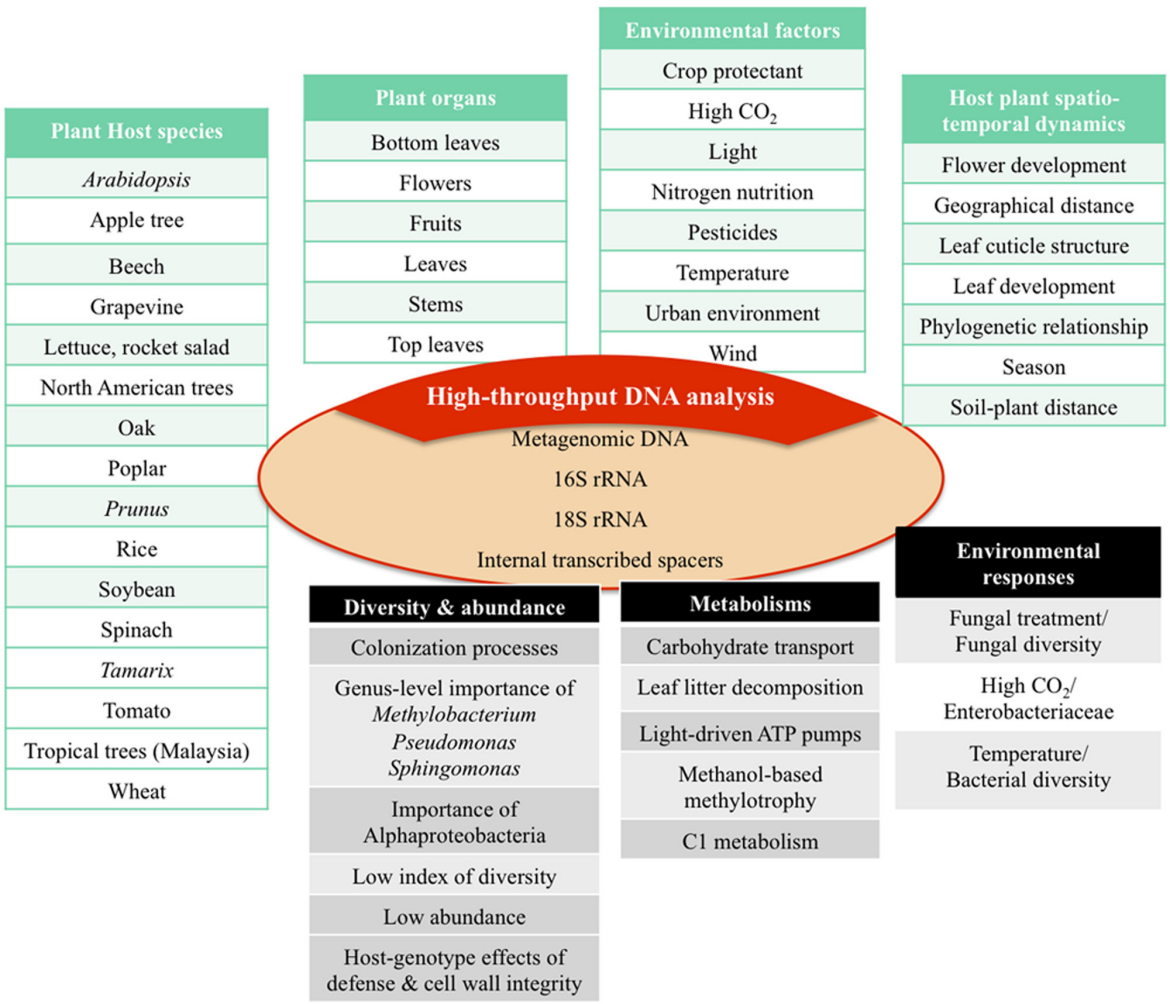
**High-throughput molecular analysis of microbial communities of the phyllosphere: from direct DNA sequencing to sequencing of prokaryotic and eukaryotic taxonomical markers**. The present range of published studies covers a significant number of plant host species in various developmental and spatio-temporal contexts and under the influence of different environmental factors. The articles presented in Supplementary Table S1 represent a selection of studies that were performed in the last years using NGS technologies. Further studies can be found in tables of other recent reviews (Rastogi et al., 2013; Knief, 2014; Müller and Ruppel, 2014). Taken together, these studies give a dynamic view of phyllosphere microbiota biodiversity, metabolisms, and environmental plasticity.

The structural analysis of phyllosphere microbial communities (Ruinen, 1961; Hirano and Upper, 2000; Schreiber et al., 2004) has been deeply renewed by the development of culture-independent mass sequencing in a growing number of plant species and cultivars of agricultural or ecological interest (**Figure 2**). Supplementary Table S1 gives a list of phyllosphere microbiota that have been characterized by high-throughput molecular analysis and summarizes the main findings of these studies. It must be noted that up to now most of these studies are based mainly on sequencing of PCR-amplified DNA-level conserved taxonomic markers (16S rRNA for bacterial taxonomy; 18S rRNA and Internal Transcribed Spacer ribosomal regions for yeasts and fungi) and, less frequently, on markers of biological functions, such as key genes related to a given metabolism, to a regulatory process or to an adaptive mechanism (**Figure 2**; Supplementary Table S1). A potential bias resulting from primer design and PCR reaction conditions has been described (Mao et al., 2012). Most 16S rRNA universal primers also amplify chloroplast and mitochondrial sequences that result in less rRNA sequences of interest matched to bacteria (Santhanam et al., 2014; Jo et al., 2015). To minimize the amplification of host plant DNA, primer 799F was designed to exclude chloroplast DNA, and the mtDNA sequences can be separated from the PCR-amplified bacterial sequences by size fractionation (Chelius and Triplett, 2001). Primer 799F has become a “standard” forward primer in recent phyllosphere microbiota analysis (Redford et al., 2010; Bodenhausen et al., 2013, 2014; Horton et al., 2014; Kembel et al., 2014; Maignien et al., 2014; Perazzolli et al., 2014; Santhanam et al., 2014; Williams and Marco, 2014; Copeland et al., 2015). Nevertheless, using primer 799F leads to systematic non-detection or underestimation of a few taxa such as cyanobacteria (Chelius and Triplett, 2001). Such bias can typically be avoided by direct mass sequencing and analysis of metagenomic DNA, which has been used so far only in a few studies of phyllosphere microbiota studies (Supplementary Table S1), but which is in constant increase as a result of ever lower sequencing costs and of ever improving bioinformatics tools.

The analysis of metagenomic data from phyllosphere microbial communities (**Figure 2**; Supplementary Table S1) essentially aims to correlate taxonomic composition (Which species is present? «Who is there?») and community structure (How abundant is each taxon? «How many are there?») with intrinsic features of the host plant (genotype, anatomy, metabolism, life history), with environmental features (geography, climate, season, pollutant exposure, phytosanitary treatments), or even with the evolutionary history of the plant species or of the plant population (domestication; relocalization). Specific studies have already shown that the assemblage of microbial communities in the phyllosphere is more similar in genetically related plants than in very divergent plant species (Redford et al., 2010; Kim et al., 2012; Bálint et al., 2013; Dees et al., 2015). Nevertheless, the spatial proximity between plants can also contribute to the composition of phyllospheric microbial communities (Finkel et al., 2012; Rastogi et al., 2012). Climatic factors such as temperature, seasons, occasional exposure to sand storms (Cordier et al., 2012; Rastogi et al., 2012; Bálint et al., 2013), or anthropogenic factors such as the use of pesticides (Shade et al., 2013; Karlsson et al., 2014; Ottesen et al., 2014; Glenn et al., 2015), play an important role in community structuration. Finally, anatomical location, whether on top leaves, bottom leaves nearer to the soil, flowers, fruits, or stems, strongly influence the structure of associated microbial communities (Ottesen et al., 2013; Shade et al., 2013).

The identification of generalist communities, usually present in phyllospheres of a given plant taxon, and specialized communities, adapted to a particular type of phyllospheric environment, is essential to achieve better understanding of the phyllosphere ecosystem and of functional interactions between plants, microbiota, and environmental features. Combinations of metagenomic and metaproteomic data have thus contributed to define the first catalogs of phyllosphere-associated generalist bacterial phyla present in different plant species, thus highlighting the involvement of Bacteroidetes, Actinobacteria, and Proteobacteria (Delmotte et al., 2009; Redford et al., 2010; Lopez-Velasco et al., 2011; Kim et al., 2012; Rastogi et al., 2012; Supplementary Table S1). In the case of yeasts, the prevalence of the phylum *Ascomycota* has been associated with microbiota from oak leaves in Europe (>90% of sequenced amplicon markers) and in Northern America, with however, significant differences of assemblages at the species level (Jumpponen and Jones, 2010; Voříšková and Baldrian, 2013).

Finally, it must be highlighted that these catalogs of phyllosphere microbial communities are magnifications and snapshots of spatially structured and highly dynamic communities. Complementary approaches such as *fluorescence in situ hybridization* (FISH) are therefore required to understand the structure and diversity of phyllospheric communities in the context of microscale spatio-temporal distributions (Remus-Emsermann et al., 2014).

## Processes of Recognition, Adhesion, and Colonization in the Phyllosphere

The cuticle is the exogenous wax layer of aerial plant surfaces which is the habitat of epiphytic bacteria and a barrier for invasive microorganisms. It is composed of long-chain fatty acids with additional pentacyclic triterpenoids and sterols and represents up to 15% of leaf dry weight (Eglinton and Hamilton, 1967). The composition of this highly lipophilic micro-structured wax layer shapes the associated phyllosphere bacterial communities as recently demonstrated using amplicon sequencing of the bacterial communities of a set of *Arabidopsis thaliana cer* mutants with different mutations in the cuticular wax biosynthesis pathways (Reisberg et al., 2013). “Plant-line-specific” bacterial communities, either positively or negatively affected by the wax phenotype, represented less than one third of the total sequence counts. “Permanent” residents, corresponding to bacterial communities that were not influenced by the wax phenotype, were affiliated with *Flavobacteriaceae*, *Flexibacteriaceae*, *Methylobacteriaceae*, *Rhizobiaceae*, *Sphingomonadaceae*, *Enterobacteriaceae*, and *Pseudomonadaceae*. Following outdoor growth, the resident bacterial community acquired as many as 2–7 bacterial clades for the wax mutant variant, unlike the wild-type plant, which was specifically enriched by only a single clade (Reisberg et al., 2013). The use of a gnotobiotic microbial community in relation with *A. thaliana* mutants has also revealed that genetic determinants of cuticle formation affected the dynamics of phyllosphere microbiota (Bodenhausen et al., 2014). All of this strongly suggests that the cuticular wax properties shape niches for specific adapted bacterial communities.

Among specific communities that are found in the phyllosphere of some plant species, the example of the *Massilia* genus is noteworthy. It is associated with lettuce leaves (Rastogi et al., 2012) and represents 7% of total bacterial population in the microbiome of spinach leaves (Lopez-Velasco et al., 2011). However, it has also been identified as a major contaminant of an aerosol with applications in agriculture, thus suggesting, as emphasized by Rastogi et al. (2012), that phyllosphere-associated *Massilia* bacteria stem from agricultural practices.

The endophytic microorganisms of the phyllosphere may be thought to be leaf epiphytic bacteria that cross the cuticle and superficial tissue layers (**Figure 1**) or endophytic bacteria that migrate from the roots. This question was addressed by sequencing 16S RNA amplicons of the epiphytic and endophytic bacterial communities associated to roots and leaves of *A. thaliana* (Bodenhausen et al., 2013). In the epiphytic communities of the phyllosphere, bacterial richness was found to be lower compared to that of endophytic communities. The richness of bacterial endophytes in both the phyllosphere and the roots was similar, with higher abundance of *Burkholderiales*, *Actinomycetales,* and *Actinoplanes* than found in the leaf epiphytic communities. These observations suggest that leaf microbial endophytes would more likely result from migration of root endophytic microorganisms within the plant than from colonization of bacteria initially present on the surface of the leaf. Nevertheless, this does not preclude foliar entrance of endophytic microorganisms, as has been shown experimentally in other cases of host–endophyte systems (Hartley et al., 2015).

## Metabolic Dynamics of Phyllosphere Microbiota

Flavobacteria are found in high abundance in the rhizosphere and phyllosphere of terrestrial plants such as *A. thaliana* where it is one of the most dominant genera of the leaf microbiota (10%; Bodenhausen et al., 2013). Flavobacteria might be highly adapted to plant carbohydrate metabolism as recently deduced from genome comparison of Flavobacteria isolated from aquatic environments and from plants. Only the genomes of Flavobacteria from terrestrial plant communities and not from aquatic communities harbored genes encoding glycoside hydrolase families GH78 and GH106, that are responsible for utilization of rhamnogalacturonan, which is exclusively associated with terrestrial plant hemicelluloses (Kolton et al., 2013).

Phyllosphere-associated microorganisms live in a sunlight-exposed habitat. Photochemical conversion of this light resource into carbon and energy that may complement carbon resources from the host plant could be a major advantage for growth in a nutrient-limited environment. Analysis of metagenomic data has revealed the presence of bacterial rhodopsin genes in phyllospheric communities (Atamna-Ismaeel et al., 2012a). It thus seems that some epiphytic microorganisms possess retinal-dependent rhodopsin proton pumps that can be light-activated by radiations covering a span of wavelengths that are distinct from the absorption spectrum of chlorophylls and carotenoids, which drive plant photosynthetic processes and thus production of the plant carbon resources that are eventually available to epiphytic microorganisms (Atamna-Ismaeel et al., 2012a; Stiefel et al., 2013).

Given the roles of carbon and nitrogen resources in nutrient signaling and nutrient regulation affecting light-dependent processes (Tolonen et al., 2006; Moran and Miller, 2007), it is likely that several metabolic pathways of epiphytic bacteria can be influenced by the carbohydrate and nitrogen status of the host plant, and thus *in fine* by the fluctuations of plant–light interactions and photoassimilate production in the host plant (Athanasiou et al., 2010; Sulmon et al., 2011). Manching et al. (2014) have recently described global links between plant nitrogen balance and leaf epiphytic bacterial species richness in maize. In a free air CO_2_ enrichment experiment, Ren et al. (2014) have also demonstrated important changes of phyllosphere bacterial communities in rice subjected to various combinations of elevated CO_2_ and different levels of nitrogen fertilization. Conversely, enzymatic activities of phyllospheric microorganisms appear to act on important plant metabolites (Huang et al., 2014), thus raising the possibility of complex feedback metabolic loops between plant tissues and phyllospheric microorganisms.

A parallel study investigated the potential presence of gene markers associated with aerobic anoxygenic phototrophic bacteria (Atamna-Ismaeel et al., 2012b). Homologs of *bchY*, which encodes the Y subunit of chlorophyllide reductase, and of *pufM*, which encodes the M subunit of the photosynthetic reaction center, have been detected in five different metagenomes from phyllosphere microbiota (rice, soybean, tobacco, tamarix, clover). Epifluorescence microscopy was used to detect the presence of specific pigments associated with aerobic anoxygenic phototrophic bacteria. It was thus found that these bacteria accounted for 1–7% of the total community of epiphytic bacteria, with the presence of the genus *Methylobacterium*, and more surprisingly, with the presence of an unknown group of bacteria, that seem to be specific to the phyllosphere (Atamna-Ismaeel et al., 2012b).

Rarefaction curves using ribosomal genes indicates that bacterial diversity in the phyllosphere is similar in several different plant species and would be in the range of human microbiome diversity. However, phyllosphere bacterial diversity seems to be much lower than those of the rhizosphere, soil or marine ecosystems (Delmotte et al., 2009; Knief et al., 2012). Sequencing depth is a significant limitation for the detection of phyllosphere-specific bacterial communities, especially in the case of low-abundance species. Thus, markers of aerobic anoxygenic phototrophic bacteria communities, which show low-abundance (<0.4%), were not detected in metagenomic data of tamarix leaf microbiota (Atamna-Ismaeel et al., 2012a), whereas direct microscopic observation revealed their presence in a number of plant species (Atamna-Ismaeel et al., 2012b). It is therefore clear that complementary approaches ought to be developed in order to detect low-abundance bacterial species in the phyllosphere, especially through microscope observation methods, such as FISH or *fluidic force microscope* approaches (FluidFM; Stiefel et al., 2013). Complementary approaches, especially through combined meta-analysis of proteomics, transcriptomics, and metabolomics data (Delmotte et al., 2009; Knief et al., 2011, 2012), are also necessary to address the nutritional and functional mechanisms of microbial adaptation to life in the phyllosphere, such as the potential involvement of auxotrophic relationships and the potential dependence of phyllospheric microbial community structure on light availability and therefore foliage and canopy stratification.

## Impact of Phyllospheric Microorganisms on Plant–Plant, Plant–Insect Herbivory, and Plant-Atmosphere-Chemical Exchanges

**FIGURE 3:**
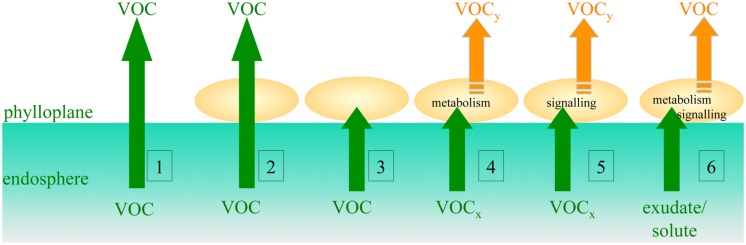
**Theoretical scenarios of the fate of phyllosphere-emitted volatile organic compounds (VOCs)**. Green and orange arrows respectively represent plant organic compound fluxes and VOCs fluxes generated from microbial epiphytes. (1) Free transfer through cuticle; (2) free transfer through cuticle and epiphytes; (3) interception by epiphytes via abiotic or metabolic processes with no VOC release; (4) biotransformation of plant VOCx by phyllospheric microbial metabolism resulting in emission of a microbial VOCy; (5) signaling by plant VOCs triggers the phyllosphere microbiota to produce a VOCy; (6) phyllosphere microbiota emit VOCs after exposure to plant non-volatile compounds. Similar scenarios involving endophytic microorganisms could also be envisaged.

**FIGURE 4:**
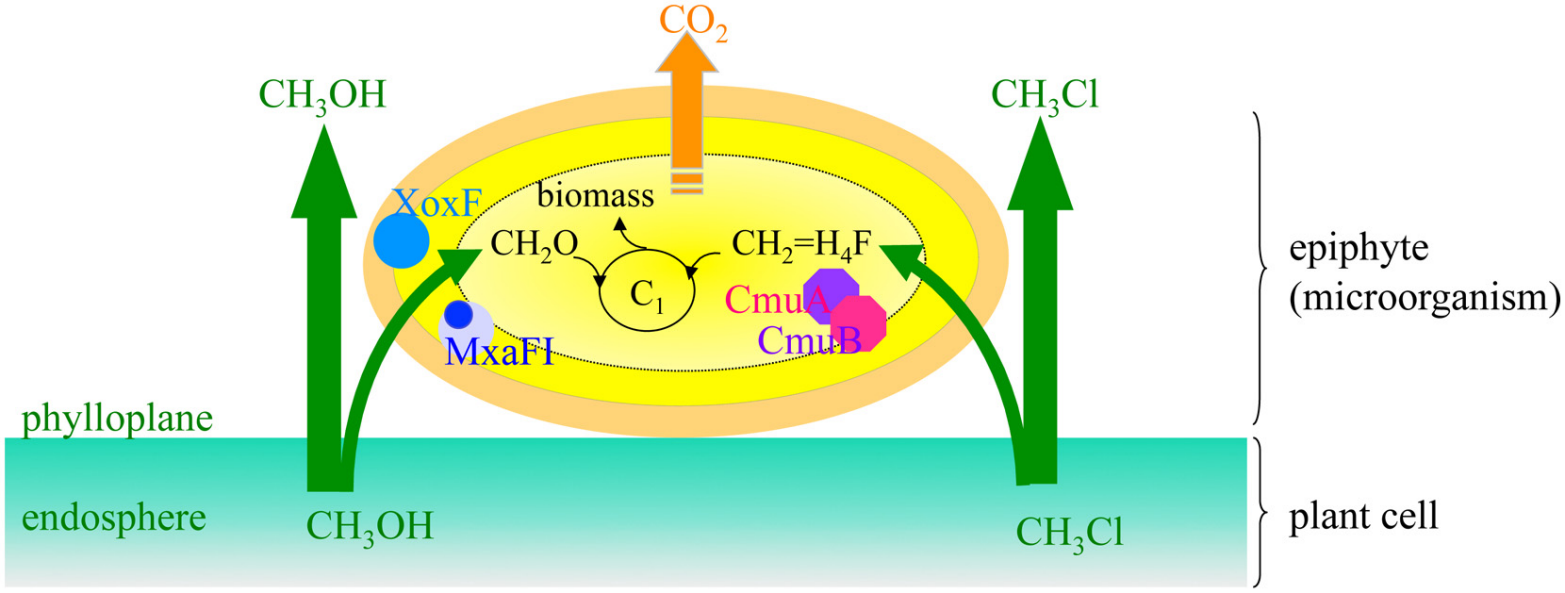
**Biocapture of plant C1 VOCs by methylotrophs of the phyllosphere**. Key enzymes in methanol oxidation imply the MxaFI methanol dehydrogenase subunits (Chistoserdova et al., 2009), and a methanol-dehydrogenase-like protein XoxF whose mutation renders the bacteria less competitive than the wild-type *Methylobacterium extorquens* AM1 during colonization of the phyllosphere of *Arabidopsis thaliana* (Schmidt et al., 2010). Unlike methanol, chloromethane oxidation by the chloromethane utilization *cmu* pathway enters the C1 central metabolism as methylene-tetrahydrofolate (CH_2_ = H_4_F) and not as CH_2_O (Vannelli et al., 1999). Bacterial isolates of surface leaves of *A. thaliana* harbor the *cmu* genes (Nadalig et al., 2011).

Plants emit a great variety of VOCs that can promote or inhibit specific species and thus contribute to numerous biotic interactions and to the shaping of microbial communities. On the other hand, microbes can intercept or alter scent emissions by plants and subsequently plant signaling with other plants or animals (Shiojiri et al., 2006). Knowledge on plant surface microbiota can reveal the mechanisms that govern processes at the interface between plants, microorganisms and plant-interacting organisms, or between plants, microorganisms, and the atmosphere (**Figures 3** and **4**), either in pristine environments, or in agricultural or anthropogenic environments.

Major molecular regulations of plant responses to abiotic and biotic challenges rely on a diverse array of phytohormones, such as the gaseous hormone ethylene, the oxylipin hormone jasmonate and its volatile derivative, methyl jasmonate, that are induced by many herbivores, and the phenolic hormone salicylate and its volatile derivative, methyl salicylate, that are induced by many bacterial pathogens. Genetic analysis of *A. thaliana* mutants has revealed a link between the community composition of phyllosphere microbiota and plant ethylene signaling (Bodenhausen et al., 2014). Using a collection of 196 recombinant inbred lines of field-grown *A. thaliana*, genome-wide association study of associated leaf microbial community revealed that plant loci involved in defense such as reproduction of viruses and cell wall integrity, trichome branching, and morphogenesis shape microbial species richness of leaf microbiota (Horton et al., 2014). In another study focusing on tobacco plants, deficiency in the phytohormone jasmonic acid biosynthesis had no detectable effect on structuring the bacterial communities (Santhanam et al., 2014). Besides phytohormones, a myriad of plant defense and signaling chemicals, whether volatile or non-volatile, are involved in plant biotic interactions (Mason et al., 2014). Plant foliage-associated bacteria have been shown to degrade plant defense chemicals, thus resulting in reduced defense against insect defoliators (Mason et al., 2014). Bacterial symbionts of the genera *Stenotrophomonas*, *Pseudomonas*, and *Enterobacter*, when secreted by the Colorado potato beetle larvae on plant surfaces, suppress the anti-herbivore defenses of tomato by enhancing the microbial defense response and thus favor larval growth (Chung et al., 2013). In a recent study of interactions between a specialist chewing insect herbivore and its sole plant host, *Cardamine cordifolia*, experimental bacterial infections of the phyllosphere showed that individual *Pseudomonas* spp. strains promoted host choice by herbivores, and that bacterial strains exhibited variation in the way they ecologically impacted insect herbivores (Humphrey et al., 2014). As described above, pesticides have strong effects on community composition in the phyllosphere (Karlsson et al., 2014; Ottesen et al., 2014; Glenn et al., 2015), thus suggesting that pesticide treatments could interfere with natural interactions between phyllosphere microbiota and plant defenses. Better understanding of defense mechanisms involving multiple biotic interactions and phyllospheric bacteria may therefore result in novel pesticide usage in the context of sustainable agriculture.

Epiphytic microorganisms present the metabolic potential for degrading compounds that are toxic to plants, to humans or to the environment. Such detoxification potential could be recruited to carry out phyllosphere-based depollution processes. This phylloremediation can target organic compounds that are already known to be metabolized by epiphytic microorganisms, such as nicotin (Sguros, 1955), phenol (Sandhu et al., 2007), polycyclic aromatic hydrocarbons (acenaphthylene, acenaphthene, fluorene, phenanthrene) which are produced by car exhausts (Yutthammo et al., 2010), or chloromethane and isoprene, which are mainly emitted by plants, and are likely to affect ozone abundance in the atmosphere (Nakamiya et al., 2009; Nadalig et al., 2014).

It has thus been shown that, in the phylum of Actinobacteria, the *Arthrobacter* genus, which is able to degrade numerous organic compounds, can grow and remain in the phyllosphere (Scheublin and Leveau, 2013). Various species of *Arthrobacter* degrade aromatic hydrocarbons (phenol, chlorophenol, BTEX, phenanthrene), *s*-triazines (atrazine, cyanazine), and various other pesticides (phenylurea herbicides, glyphosate, malathion; Scheublin and Leveau, 2013).

Using custom-made microarrays of the Gram-positive *Arthrobacter* with species members commonly found in epiphytic bacterial communities (Rastogi et al., 2012), comparative transcriptome profiling with bacteria recovered from leaves of the common bean (*Phaseolus vulgaris*) or from growth on agar surfaces, demonstrated that several *cph* genes involved in 4-chlorophenol degradation had phyllosphere-induced expression (Scheublin et al., 2014), most likely resulting from the presence of natural plant-excreted phenolic compounds. The utilization of plants harboring adequate microbial communities that degrade a given set of organic compounds can be envisaged for processes of atmospheric depollution in urban or industrial environments, and for the depollution of atmospheric drifts of phytosanitary products in agricultural environments. Finally, it can also be envisaged that epiphytic microorganisms that have beneficial effects on plants could be used as probiotic agents (Berlec, 2012).

## Impact of Phyllospheric Microorganisms on Plant- Atmosphere-Climate Interactions

Plants emit a number of VOCs or VOC precursors that are transferred through the phyllosphere and that probably play a role in climate regulation (Otte et al., 2004; Peñuelas and Staudt, 2009; Schäfer et al., 2010). In the biosphere, plants are the main source of VOC emissions amounting to more than 1,000 Tg year^-1^, with components as diverse as terpenes, monoterpenes and C_1_ compounds, including methanol, methane, and halogenated methane. What is known of how plant emissions of VOCs interact at their surface with bacterial epiphytes has recently been reviewed (Junker and Tholl, 2013). It remains largely unknown how and to what extent VOCs emitted by plants could be biocaptured, intercepted or consumed through bacterial metabolism by epiphytes present directly on the surface of plants (**Figure 3**), or by transiently occurring airborne bacteria, and how the effects of climate change will impact the abundance, diversity, and ability of microbial metabolism in filtering of plant-emitted VOCs.

Methylotrophic microorganisms are able to utilize some of the plant organic compounds containing a single carbon atom or lacking C–C bonds such as methanol (CH_3_OH), formaldehyde (CH_2_O), and chloromethane (CH_3_Cl). Methylotrophic microorganisms are ubiquitous and can be found in roots and leaves of plants (Delmotte et al., 2009; Knief et al., 2012; Jo et al., 2015), and in the air (DeLeon-Rodriguez et al., 2013). A prominent C_1_ source for epiphyte microbiota (**Figure 4**) is methanol that has been proven to confer an advantage *in situ* to methylotrophic epiphytes such as the Alphaproteobacteria *Methylobacterium extorquens* and the methylotrophic yeast *Candida boidinii* (Sy et al., 2005; Kawaguchi et al., 2011). Seedlings of *Nicotiana* emitted methanol at 0.005 to 0.01 ppbv in the presence of *M. extorquens*, while plants not colonized by these bacteria showed much higher emissions (0.4–0.7 ppbv; Ababda-Nkpwatt et al., 2006).

Methane (CH_4_) is the most abundant organic trace gas in the atmosphere (with a mixing ratio of ∼1.8 ppm) and an important greenhouse gas. Both intact plants and detached leaves emit methane at an initial estimated source strength of 62–236 Tg/year for living plants and 1–7 Tg/year for plant litter (Keppler et al., 2006). Plants internally transport methane to the atmosphere through the roots, stems, and leaves from the rhizosphere, where plant exudates provide the nutrients for growth of methanogenic bacteria. Pathways of direct methane production by plant tissues also exist (Althoff et al., 2014; Lenhart et al., 2015). Methane emissions rates depend on plant species and on abiotic factors such as the water regime and temperature (Bhullar et al., 2013). Moreover, directly at the phylloplane level, plant methane emissions would result from wax degradation, in addition to the previously suggested pectin degradation, in the presence of UV radiation and oxygen (Bruhn et al., 2014). Methanotrophic bacteria that utilize methane as a source of carbon and energy have been found in the phyllosphere of plants (Iguchi et al., 2012).

Isoprene (2-methyl-1,3-butadiene) is emitted by leaves of many plant species and emission was shown in some cases to increase with higher temperatures (Monson et al., 1992). The magnitude of global isoprene emissions to the atmosphere is similar to that of methane, and isoprene is an important precursor for photochemical ozone production when oxides of nitrogen levels are high (Arneth et al., 2008). A bacterial degradation pathway has been genetically characterized in a marine isolate, *Rhodococcus* sp. strain AD45 (van Hylckama Vlieg et al., 2000), but has not so far been demonstrated in plant isolates.

Chloromethane (CH_3_Cl; methyl chloride) is the most abundant chlorinated organic compound in the atmosphere (currently ∼550 ppt) and is considered to be responsible for over 16% of the halogen-catalyzed depletion of stratospheric ozone (World Meteorological Organization, 2014). In *A. thaliana*, chloromethane is the product of *S*-adenosylmethionine-dependent methylation of chloride, which is catalyzed by a protein encoded by the *HOL* (*HARMLESS TO OZONE LAYER*) gene, although a physiological *in planta* role for enzyme-produced chloromethane remains to be demonstrated (Nagatoshi and Nakamura, 2009). To assess if vegetation is the main contributor to global emissions of chloromethane to the atmosphere, a fluorescence-based bacterial bioreporter for chloromethane detection has been developed and validated in the model chloromethane-producing plant *A. thaliana* (Farhan Ul Haque et al., 2013). Bacterial adaptation to growth on chloromethane as the sole source of carbon and energy by the *chloromethane utilization* (*cmu*) pathway has been characterized in *M. extorquens* CM4 (Roselli et al., 2013). So far, the few cultivable chloromethane-degrading strains isolated from plants, which were affiliated to the genus *Hyphomicrobium* (Nadalig et al., 2011), were also degrading methanol, thus being able to filter several C1 VOCs emitted on plant leaf surfaces (**Figure 4**).

Volatile dimethyl sulphide (DMS) is considered to be an important global-climate regulator (Charlson et al., 1987; Schäfer et al., 2010; Nevitt, 2011). Fluxes and dynamics of DMS are strongly associated with oceanic sulfur cycles and with phytoplanktonic production of the DMS precursor dimethylsulphoniopropionate (DMSP; Charlson et al., 1987; Schäfer et al., 2010; Nevitt, 2011). However, some plant species (Otte et al., 2004), small in numbers, but ecologically significant, such as salt marsh grasses of the genus *Spartina* and sugar canes (*Saccharum* sp.), are efficient producers of DMSP, which can be metabolized to acrylate and DMS by plant-associated microbes possessing DMSP lyase (Ansede et al., 2001). Phyllosphere microbiota could therefore act on plant-related DMS dynamics both through DMSP-DMS transformation and through DMS metabolization. It is therefore highly likely that phyllosphere microbiota play major roles in carbon and sulfur biogeochemical cycles, in ecosystemic signaling and in climate regulation through their action on plant-related volatile compounds, thus requiring that understanding of the functional ecology of phyllospheric microbes, especially in species of the *Spartina* and *Saccharum* genera, is improved.

**FIGURE 5:**
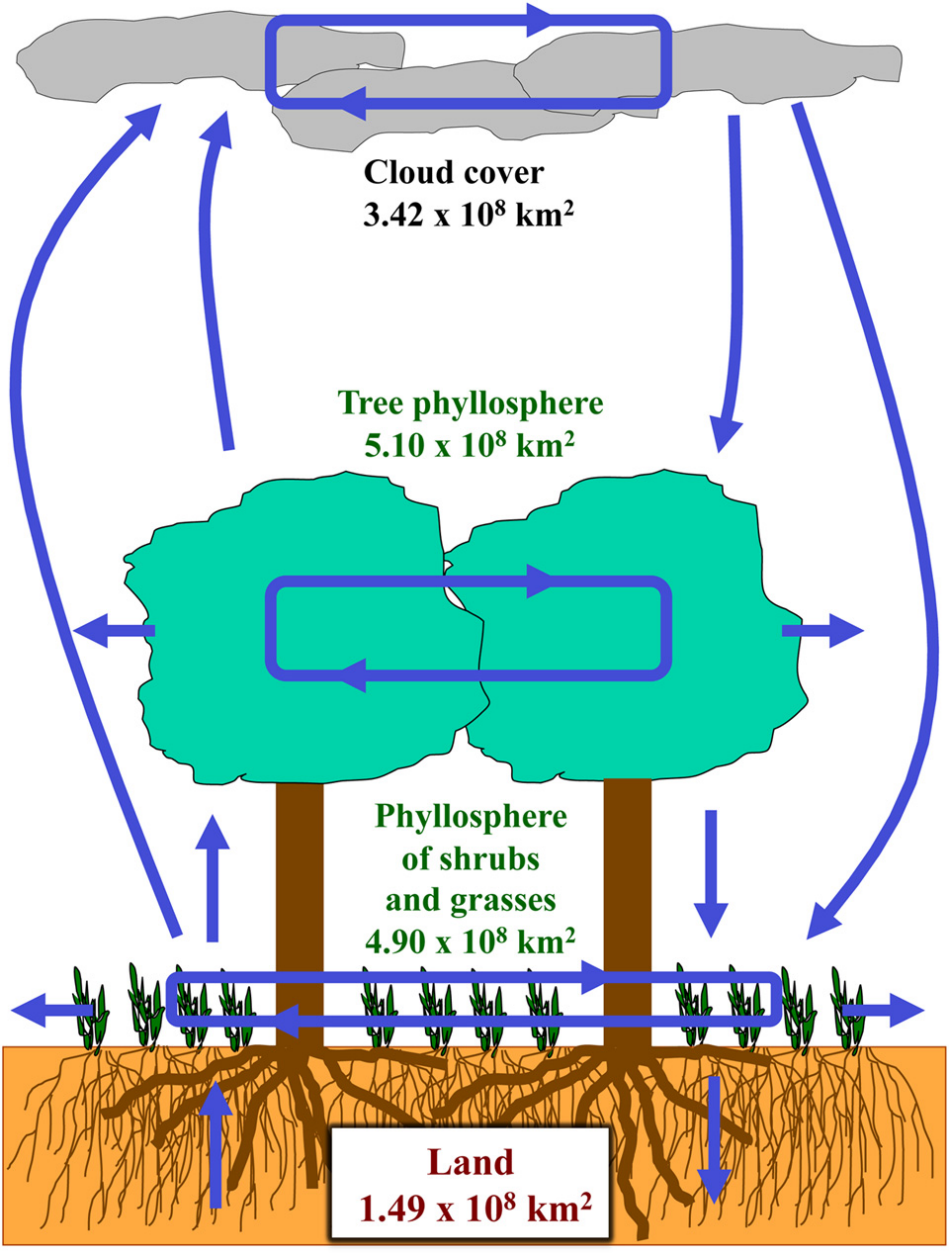
**Dynamics of interactions of plant canopies and the atmosphere**. Land, plant strata, and cloud strata are represented, with estimates of global earth-wide surfaces, which were derived from recent studies (Friend, 2010; Probst et al., 2012; Vorholt, 2012). Interactions involving fluxes of microbial populations are symbolized by blue arrows (Lindemann et al., 1982; Finkel et al., 2012; Rastogi et al., 2012; DeLeon-Rodriguez et al., 2013; Šantl-Temkiv et al., 2013; Vaïtilingom et al., 2013; Hill et al., 2014).

Pioneering studies on microbiota in clouds have shown the presence of prevalent bacteria that are common with phyllosphere microbiota, thus suggesting that at least some epiphytic microorganisms are adapted to the conditions of the troposphere (DeLeon-Rodriguez et al., 2013; Šantl-Temkiv et al., 2013). Tropospheric microorganisms are likely to act as water condensation or nucleation centers during cloud formation and to be involved in global carbon cycles through metabolization of the organic compounds that are present in clouds (Vaïtilingom et al., 2013). Moreover, epiphytic microorganisms may constitute the major source of airborne bacteria, including ice nucleation-active (INA) bacteria. These bacteria mainly belong to the order of Gammaproteobacteria and possess common INA proteins encoded by *ina* genes that were qPCR quantified and estimated to reach up to 10^8^ *ina* genes per g of fresh weight in the foliage of cereals (Lindemann et al., 1982; Hill et al., 2014). The presence of INA proteins may also contribute to bacteria dissemination processes via deposition on cloud droplets. There may thus be strong links between phyllosphere microbiota and cloud microbiota (**Figure 5**) with important implications for climate regulation.

The combined activities of microbial communities in the phyllosphere, through the emission of VOCs and interactions with plant VOCs (**Figure 3**), through complex phyllosphere–atmosphere exchanges (**Figure 5**), and through ice-nucleation processes, are therefore potential mechanisms of major global impact on the biosphere. As described above, the composition of phyllosphere microbiota have an impact not only on plant growth and plant health, but also on plant-derived greenhouse and ozone-depleting gasses. Conversely, global change parameters such as elevated CO_2_ and limited nitrogen have an impact on the composition of phyllosphere microbiota, at least through indirect effects on plant growth and metabolism. Such a network of see-saw relationships may thus be the basis for complex positive or negative feedback loops that could enhance or refrain global change processes.

## Novel Perspectives for Molecular, Physiological, and Ecological Studies of Phyllospheric Plant-Microorganism- Atmosphere Interactions

Current research is at the start of the characterization of terrestrial phyllosphere microbiota, and is likely to open new perspectives in microbial ecology, in plant ecophysiology, and in environmental sciences, that will go beyond the agronomical framework which, up to now, has been mostly taken into consideration. As is the case for numerous studies of microbial communities (Vandenkoornhuyse et al., 2010), phyllosphere metagenomics is greatly expanding this field of microbial ecology which covers a vast terrestrial compartment that was rather neglected.

Major issues of functional ecology related to phyllospheric microbial communities have already been identified and their potential importance requires a major effort of future research: (i) To what extent are epiphytic microorganisms involved in the chemical composition of the atmosphere through their potential action on gaseous molecules synthesized and emitted by plants? (ii) To what extent are epiphytic microorganisms able to act on toxic volatile products of anthropogenic origin? (iii) To what extent are phyllosphere microbiota interacting with microbe-associated molecular patterns triggering innate immunity in plants (Newman et al., 2013) and thus involved in the health and protection of plants? (iv) What is the impact of phyllospheric bacteria on gut microbial composition of herbivorous animals (Charrier et al., 2006; Hehemann et al., 2010; Thomas et al., 2011; Priya et al., 2012)?

Finally, in the context of land use and climate global changes (Peñuelas and Staudt, 2009), the complex dynamics between plants, phyllosphere microbiota, trace gasses, atmosphere microbiota and climate processes (**Figures 3–5**) urgently needs further investigation and, in particular, modeling analysis of potential regulatory feedback loops.

The dynamics of phyllosphere communities is also well-adapted as a model for studies in theoretical ecology concerning the origin of biodiversity, biotic interactions and community assemblage mechanisms (Finkel et al., 2012; Meyer and Leveau, 2012). It is thus bound to raise novel issues in evolutionary ecology, such as the co-evolutionary links between phyllosphere microbiota and host plants, and the possibility of symbiotic interactions in the phyllosphere, and especially on the phylloplane. However, the ongoing microbial colonization of plant surfaces and the ongoing sweeping of bacteria from plant surfaces (**Figure 5**) is likely to result in complex kinetics of plant–microorganisms interactions in the phyllosphere. Moreover, these interactions are likely to cover a wide range of affinities from loose associations to intimate symbioses. The complexity and the fluctuations of these interactions therefore entail that direct application of the holobiont concept (Zilber-Rosenberg and Rosenberg, 2008) to any kind of plant-phyllosphere system must be taken with caution, and that plant-phyllosphere systems should be better described by a fuzzy holobiont concept.

All of these novel and recent results and issues are the topics of active discussions and commentaries in the field of environmental microbiology. It is an effervescent field where further studies of phyllosphere microbiota are actively encouraged among environmental microbiologists by funding schemes at an international level. However, the importance of phyllosphere microbiota for plant functioning at physiological and ecological levels, the idiosyncracies of plant molecular mechanisms, and the complex regulatory loops between plants, microorganisms and the atmosphere advocate for the intensification of collaborations between plant scientists, biogeochemists and microbiologists.

## Conflict of Interest Statement

The authors declare that the research was conducted in the absence of any commercial or financial relationships that could be construed as a potential conflict of interest.
