# Supplementary material for: Pivotal roles of phyllosphere microorganisms at the interface between plant functioning and atmospheric trace gas dynamics
Source: Front Microbiol. 2015 May 22;6:486. doi: 10.3389/fmicb.2015.00486 (PMC4440916; doi:10.3389/fmicb.2015.00486)
Supplement: Supplementary file 1 [file Table_1.DOC]

| **Supplemental data Table S1**  High throughput molecular analysis of microbial communities of the phyllosphere: from direct DNA sequencing to sequencing of prokaryotic and eukaryotic taxonomical markers | | | |
| --- | --- | --- | --- |
| **Plant-host** | **Massive sequencing target** ^a^ | Conclusions Citations | |
| Soybean (*Glycine max)* | metagenomic DNA (261 Mbases) | Metagenome of soybean leaf phyllospheric microbiome performed in the elegant and original framework of a functional proteogenomic study of leaves of soybean, clover (*Trifolium repens*) and of the model plant *Arabidopsis thaliana*. For the three plants, the taxonomical data based on DNA and proteins are consistent. Two genera of the Alphaproteobacteria are predominantly encountered: *Methylobacterium* expresses proteins involved in plant-produced one-carbon compounds utilization as the sole source of carbon and energy for growth; *Sphingomonas* abundantly expressed TonB-dependent receptors involved in various carbohydrate transport processes suggesting a large substrate utilization pattern for adapted growth on leaf surfaces. | Delmotte *et al*., 2009 |
| Rice cultivar (*Oryza sativa*) | metagenomic DNA (831 Mbases)  16S rRNA (V4 region) | Comparative metagenome and metaproteomic analysis of the microbiomes of the phyllosphere and the rhizosphere. The microbiome complexity and composition associated to the cultivar IR-72 is different from that of two other cultivars (PSB, RC80). Predominant communities of the phyllosphere are affiliated to Alphaproteobacteria *(Methylobacterium,* and *Rhizobium/Agrobacterium*) and to Actinobacteria. Methanogen and methanotroph metabolism-associated proteins were found in the rhizosphere metaproteome, whereas methanol-based methylotrophy linked to the genus *Methylobacterium* dominated within the phyllosphere metaproteome.  The structure and composition of phyllosphere bacterial communities was mostly affected by rice growth stage (tillering, filling, and maturity stages) rather than by CO_2_ and N treatments. Predominant communities of the phyllosphere are affiliated to Proteobacteria, with 88.5% affiliated to Gammaproteobacteria. Elevated CO_2_ was found to increase *Enterobacteriaceae* from 89% at ambient CO_2_ to 97% at elevated CO_2_ under low N fertilization at the filling stage. | Knief *et al*., 2012  Ren *et al*., 2014 |
| Tamarix (*Tamarix lotica*) | metagenomic DNA (448 Mbases) | Metagenome of leaf microbiome is compared to metagenomes of microbiomes associated to other plants and habitats (soils, fresh and marine water): evidence of the ability of epiphytes to express rhodopsin on the leaf surface and enable solar radiation utilization as an energy source. | Atamna-Ismaeel *et al*., 2012a |
| Tomato (*Solanum lycopersicum*) | metagenomic DNA^b^ (6 Gbases) and 16S rRNA, 18S rRNA  16S rRNA (V4 region) and 18S rRNA | Differences in microbial community structure and diversity were greater between time-points compared to treatments with a crop protectant (acibenzolar-S-methyl) and with a copper pesticide. Nevertheless, the topical activity of the copper pesticide seemed to reduce levels of Gammaproteobacteria. For the Eukarya, no distinguishable differences in alpha and beta diversity between treatments were observed.  Microbiome cartography of anatomic parts within a plant (flower, fruit, stem, roots, and top and bottom leaves). Most frequently observed epiphytes for aerial parts include bacteria affiliated to *Pseudomonas* and *Xanthomonas*. Dominant fungi are *Hypocrea,* *Aureobasidium* and *Cryptococcus*. A gradient of compositional similarity correlated to the distance of a given plant from the soil. | Ottesen *et al*., 2014  Ottesen *et al*., 2013 |
| Table S1-continued | | | |
| *Arabidopsis thaliana* | 16S rRNA (V5, V6, V7 region) and fungal ITS1  16S rRNA (V5, V6, V7 region)  16S rRNA (V3, V4, V5 regions)  16S rRNA (V5,V6 regions) | The leaf microbiome of 196 genotyped variants, field-grown at a single location, had a majority of bacterial OTUs belonging to the Proteobacteria (*Sphingomonas*, *Rhizobium*, *Pseudomonas*), Bacteroidetes (*Flavobacterium*) and Actinobacteria, and of fungal OTUs belonging to the ascomycete classes Dothideomycetes and Sordariomycetes, and the basidiomycete class Tremellomycetes. For the heavily sequenced taxa, bacterial and fungal communities were demonstrated to be shaped by host genetic variations involved in similar biological processes responsible for defense and cell wall integrity.  *Pseudomonas*, *Sphingomonas* and *Methylobacterium* are more abundant in the leaves compared to the roots. In the phyllosphere, richness is lower in the epiphytic communities compared to the endophytic communities with a distinct distribution of Burkholderiales, Actinomycetales and *Actinoplanes*. These phyla are less abundant in the leaf epiphytic communities than in the leaf endophytic or root-associated communities suggesting that leaf endophytes would originate mostly from root endophyte migration rather than from leaf epiphyte internalization.  The integrity of the leaf cuticule partially structures the phylloplane communities (*cer* cuticular wax mutants compared to wild-type plants). Nevertheless, 8 OTUs within the Proteobacteria and Bacteroidetes represented nearly two thirds of the total sequence counts and were designed as “permanent” resident communities.  Phyllosphere bacterial communities are drawn from low-abundance airborne bacterial populations at early plant stage, and subsequently converge to distinctive bacterial community composition among replicates, which was found to be strongly related to the spatial association of individual plants (21 samples over 73 d). Thus, stochastic colonization and dispersion seem to play a major role in shaping the abundance structure of phyllosphere bacterial populations. | Horton *et al*., 2014  Bodenhausen *et al*., 2013  Reisberg *et al.*, 2013  Maignien *et al*., 2014 |
| *Canola (Brassica napus), common bean (Phaseolus vulgaris),* soybean (*G. max)* | 16S rRNA gene ^b^ (V5-V7 regions) | For 3 agricultural field-grown crop species in Canada, the composition of 103 leaf bacterial communities is primarily driven by temporal changes and community succession rather than plant host species. Communities sampled at the beginning of the growing season more closely resembled the soil community and, as the season progressed, the phyllosphere microbiome became increasingly distinct and less diverse. | Copeland *et al*., 2015 |
| Lettuce (*Lactuca sativa*), rocket salad (*Diplotaxis tenuifolia*) | 16S rRNA ^b^ (V4 region)  16S rRNA (V5-V6-V7 regions)  16S rRNA (V5-V9 regions) | Microbiomes of 51 field-grown plants (*L. sativa*, *D. tenuifolia*; 2 farms, Norway), in function of plant maturity (3 weeks and harvest), and season (2-3 plantings in 2013). Bacterial colonization of leaves seems to be driven by season rather than leaf maturity. At harvest, significant phyllosphere community variability was observed between plant species at the same location. Proteobacteria, Bacteroidetes, Actinobacteria, and Firmicutes were the most prevalent phyla. The predominant families were *Pseudomonadaceae*, *Oxalobacteraceae*, and *Enterobacteriaceae*. At the level of genera, *Pantoea* and *Sphingomonas* can be assigned to the bacterial core phyllosphere microbiota.  Microbiomes of 88 field-grown lettuces (*L. sativa*) in function of time (2 seasons), space (field position) and environment (two USA states). Enterobacteria are more abundant in summer than in winter. Microbiome composition variations increased with distance between fields or with the timing of a dust storm. Most-represented phyla are Proteobacteria, Firmicutes, Bacteroidetes and Actinobacteria. At the level of genera, *Pseudomonas,* *Bacillus,* *Massilia,* *Arthrobacter* and *Pantoea* were assigned to the bacterial core phyllosphere microbiota.  Microbial community transplantation on lettuce plants (*L. sativa*) grown indoors restored the field-like microbiota with significantly higher proportions of Gammaproteobacteria such as *Enterobacteriaceae* and *Moraxellaceae* families compared to laboratory-grown plants with Betaproteobacteria, such as the *Comamonadaceae* and *Burkholderiacea*. | Dees *et al*., 2015  Rastogi *et al*., 2012  Williams & Marco, 2014 |
| Table S1-continued | | | |
| Spinach (*Spinacia oleracea*) | 16S rRNA | Inventory of the phyllosphere bacterial communities following shifts in storage temperature. | Lopez-Velasco *et al*., 2011 |
| Tobacco (*Nicotiana attenuata*) | 16S rRNA (V4–V9 regions) | Developmental stages and jasmonic acid-mediated defense response signaling do not have a major effect on structuring the bacterial communities as tested on plants deficient in jasmonate biosynthesis in field and laboratory conditions. | Santhanam *et al.*, 2014 |
| Tamarix (*Tamarix aphylla*) | 16S rRNA (V4-V6 regions) | Geographical distance (2 leaf specimens for 6 sites along a 500-km east-west transect in the stable ecosystem of the Sonoran desert, USA) correlated with Betaproteobacteria occurrence within the phylloplane microbiota. | Finkel *et al.*, 2012 |
| Trees | 16S rRNA  16S rRNA (V1-V3 regions)  16S rRNA (V4 region)  16S RNA (V5-V6 regions) | The composition of bacterial communities is more similar for leaves of phylogenetically-related trees (10 species^c^ from a north American campus, except for *Pinus ponderosa* from different continents).  Each species (6 rainforest species^d^ in Malaysia) has a distinct phyllosphere bacterial community, which shared less than 1% OTUs with surrounding soil. Acidobacteria represents a predominant common phylum (17%).  Cartography of bacterial communities at 5 different stages of flower development (6 apple trees) with or without streptomycin spray. Presence of 2 preponderant phyla: TM7 and *Deinococcus*-*Thermus*.  Bacterial community structure on tree leaves (57 species in a neotropical forest in Panama) was correlated to plant traits (phylogeny, growth and mortality rates, wood density, leaf mass per area, and leaf nitrogen and phosphorous concentrations). Dominant phyla included Actinobacteria, Alpha-, Beta-, and Gammaproteobacteria, and Sphingobacteria, among an average of 400 bacterial taxa per leaf from individual trees. | Redford *et al*., 2010  Kim *et al*., 2012  Shade *et al*., 2013  Kembel *et al*., 2014 |
|  | 16S rRNA (V5-V6 regions)16S rRNA (V1-V3 regions) and 18S rRNA | Leaf bacterial communities of four *Prunus* species [apricot (*P. armeniaca*), cherry (*P. cerasus*), peach (*P. persica*), plum (*P. domestica*); single sampling; same orchard, South Korea] are dominated by *Sphingomonas* and *Methylobacterium* genera, and besides these dominant genera, host-dependent bacterial diversity was observed.  Pest management systems (synthetic versus organic using a kaolin-based insect repellent) did not develop unique apple leaf microbial populations (5 samplings over 4 years). Kaolin provided a habitat in which microbial populations were enhanced 2–5 times. Five bacteria (*Geitlerinema*, *Massilia*, *Methylobacterium*, *Sphingomonas*, *Synechococcus*) and 4 fungal (*Alternaria*, *Aureobasidium*, *Catenulostroma*, *Phoma*) genera were present in all samplings. | Jo *et al*., 2015  Glenn *et al*., 2015 |
| Balsam poplar (*Populus balsamifera*) | fungal ITS | The composition of the foliar fungal communities is structured by the plant genotype over 2 vegetative periods and 2 translocations. | Bálint *et al*., 2013 |
| Bur oak (*Quercus macrocarpa*) | fungal ITS | The fungal communities differ between urban and adjacent nonurban environments, albeit not consistently across the growing season. | Jumpponen & Jones, 2010 |
| European beech (*Fagus sylvatica*) | fungal ITS1 | The composition of phyllosphere fungal assemblages varies along an elevation gradient (temperature). | Cordier *et al*., 2012 |
| Table S1-continued | | | |
| Grapevine (*Vitis vinifera*) | fungal ITS and 16S rRNA (V5-V9 regions) | The bacterial and fungal microbiota of grapevine leaves of the cultivar Pinot gris differed more when compared at three locations of northern Italy than when treated with the chemical fungicide penconazole and the biological control agent *Lysobacter capsici*. The dominant bacterial genera *Sphingomonas*, *Janthinobacterium*, *Methylobacterium*, and *Pelomonas* varied among the grapevine locations, unlike the genera *Pseudomonas*, *Erwinia*, and *Acetobacter*, which were found in all samples. The dominant fungal phyla were *Ascomycota* and *Basidiomycota*. | Perazzolli *et al.,* 2014 |
| Sessile oak (*Quercus petraea*) | fungal ITS and gene *cbhI* | Two months before leaf drop, the mycoflore presents a diverse fungal community involved in the early process of leaf decomposition. Leaf litter harbors successive fungal communities without correlation with the observed diversity of the cellulobiohydrolase-coding gene *cbhI* which was used so far as a cellulose degradation marker. | Voříšková & Baldrian, 2013 |
| Wheat (*Triticum aestivum*) | fungal ITS2 | Commonly-used fungicide-treated and untreated samples (18 fields in 2 areas in Sweden) were compared. Fungicide use altered the relative abundance of several saprotrophs, and was negatively correlated to community evenness. The dominant fungal phyla were *Ascomycota* and *Basidiomycota*. | Karlsson *et al*., 2014 |

a, Unless specified, 454 pyrosequencing of PCR-amplified products of prokaryotic 16S rRNA-encoding genes, eukaryotic/yeast 18S rRNA-encoding genes, and /or fungal ITS region for «  Internal Transcribed Spacer  »

b, Illumina MiSeq sequencing

c, *Acer platanoides, Abies concolor, Aesculus hippocastanum, Catalpa speciosa, Celtis occidentalis, Cercis canadensis, Fraxinus pennsylvanica, Picea pungens, P. flexilis, Tilia americana*

d, *Arytera littoralis, Dillenia excelsa, Dyera costulata, Gnetum sp., Schizostachyum brachycladum, Shorea maxima*
